# Feature Learning Based Random Walk for Liver Segmentation

**DOI:** 10.1371/journal.pone.0164098

**Published:** 2016-11-15

**Authors:** Yongchang Zheng, Danni Ai, Pan Zhang, Yefei Gao, Likun Xia, Shunda Du, Xinting Sang, Jian Yang

**Affiliations:** 1 Department of Liver Surgery, Peking Union Medical College Hospital, Chinese Academy of Medical Sciences and Peking Union Medical College, Beijing, 100730, China; 2 Beijing Engineering Research Center of Mixed Reality and Advanced Display, School of Optics and Electronics, Beijing Institute of Technology, Beijing, 100081, China; Jiangnan University, CHINA

## Abstract

Liver segmentation is a significant processing technique for computer-assisted diagnosis. This method has attracted considerable attention and achieved effective result. However, liver segmentation using computed tomography (CT) images remains a challenging task because of the low contrast between the liver and adjacent organs. This paper proposes a feature-learning-based random walk method for liver segmentation using CT images. Four texture features were extracted and then classified to determine the classification probability corresponding to the test images. Seed points on the original test image were automatically selected and further used in the random walk (RW) algorithm to achieve comparable results to previous segmentation methods.

## 1. Introduction

The liver, which secretes bile, is the largest digestive gland and detoxification organ in the body. This organ frequently suffers from lesions because of its numerous functions. According to the World Health Organization, 745 thousand people have died because of liver cancer last 2015 [1]. Thus, prevention and treatment of liver disease is urgent and has become a hot topic for related research worldwide. Computed tomography (CT) imaging provides accurate anatomic structural information of the liver and its lesions [2,3]. CT images with high signal-to-noise ratio and high spatial resolution have become an important imaging modality and basis for the diagnosis and treatment of liver diseases.

Segmentation technique extracts the structure of the liver and constructs the geometrical expression of the liver shape. This method is indispensable for volume measurement, functional assessment, lesion location and operation planning [4]. The shape and size of the liver remarkably differ among individuals. Manual extraction of liver structure continues to be the primary procedure applied by clinicians, but this process is time-consuming and relies on subjective judgment. Numerous segmentation methods have been developed. The detailed overviews for liver segmentation are referred to recent and extensive reviews [5–7]. However, precise segmentation of livers remains the most challenging task in medical image processing because of the sharp corners, concave regions, and similar intensities with other organs.

Chi et al. [8] proposed an improved active contour model for liver segmentation in which template matching, K-means clustering, and snake model are combined to constrain the deformation of active contour to approach the boundary of an object. Rikxoort introduced the registration technology [9] to constrain the deformation of the model. KNN classifier was used to roughly segment the liver from the background, and accurate segmentation was achieved by B-splines [10]. Seghers used a priori knowledge of the liver boundary [11] to construct the external force constraint model. This model enhanced the local segmentation accuracy of the liver to a certain extent. However, approaching hepatic depressions and sharp corners causes the difficulty in maintaining the smoothness of livers. The validity and robustness of internal/external constraint model should be improved in the above deformation model methods.

Hufnagel et al. proposed a statistic shape model for liver segmentation [12]. Principal component analysis (PCA) was used to represent the shape of livers. Features manually extracted from the test liver were matched with those from training livers. The statistical shape model was iteratively deformed in 3D space to obtain the final segmented result. Meanwhile, Saddi used the Gauss mixture model to initialize the statistical shape model [13]. Gradient descent method was used to minimize the energy function of the level set after the initial boundary of level set was manually obtained. Afterward, the statistical shape model and level set shape were registered to decide the boundary of the liver. Kainmüller et al. built a shape constraint plane using the position relation of statistical model vertices and their neighborhood points to determine the scale and range of deformation to remedy over-segmentation. However, optimal setting of the initial contour should be improved since exact matching between prior shape models is difficult. Moreover, personalized information was not considered in building the energy of a statistical shape model, which is an indispensable factor for precise segmentation.

Furukawa et al. [14] proposed a segmentation method by comparing the maximum a posteriori estimation and level set. Probabilistic atlas was built to constrain the energy function of the level set which was driven to approach the boundary of the liver. Slagmolen et al. [15] proposed a method based on interactive probabilistic atlas registration. The average model was obtained after all training images were registered with manually selected mark points. Meanwhile, the test image was roughly segmented by the threshold method. Final segmentation result was achieved by deforming the average model after the non-rigid registration between the average model and rough segmentation. Although a relatively accurate segmentation result was obtained, probabilistic atlas is subjective and time consuming.

A lot of fully-automatic methods has been provided for liver segmentation. Based on inconsistent contrast-enhancement and spurious imaging artifacts, Marius [16] proposed an affine invariant shape parameterization for liver segmentation and refined with a geodesic active contour. Huang [17] proposed a hybrid approach by combining liver intensity range detection, atlas-based affine, non-rigid registration and shape constrained differeomorphic demons. Based on the level set framework, Wimmer [18] used boundary model, region model and shape model to avoid a parameterization of the target shape. Kainmuller [19] combined the statistical deformable model with a constrained free-form to computing the displacements and initial positioning of the model.

In this paper, a feature-learning-based random walk method (FLRW) is presented for liver segmentation using CT images. Four kinds of texture features were extracted and fused to train a hybrid classifier that used the SVMs as weak classifiers for Adaboost. A liver-specific probabilistic image for each unlabeled image was generated. Final segmentation was obtained based on a random walk combined with the generated probabilistic images. The main contributions of this work are summarized as follows: (1) a probabilistic image in order to capture the relationship between pixels is as a pre-segmentation result; (2) the texture feature rather than only intensity are used to improve the weights which were significant to the random walk for segmentation; (3) the segmentation framework is appropriate for two different databases even a few training images from one database are used.

The rest of this paper is organized as follows. After the introduction, the methodology, including probabilistic image learning and liver boundary determination, is described in Section 2. Evaluation results are presented in Section 3. Discussion and conclusions are presented in Section 4.

## 2. Methodology

**Fig 1:**
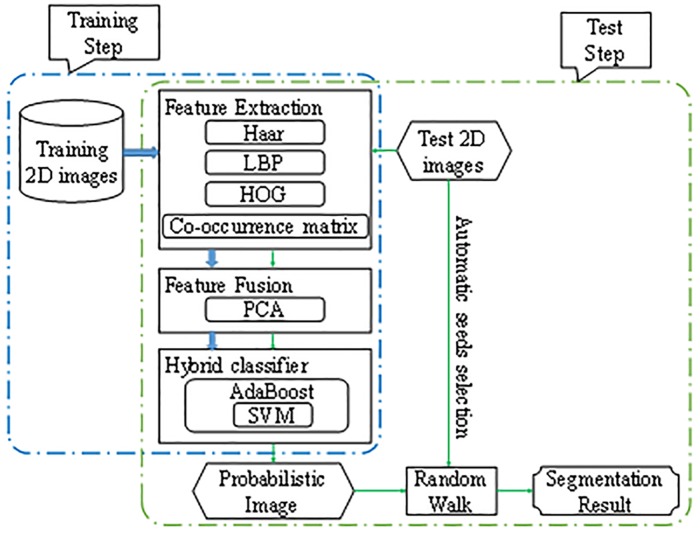
Flowchart of the proposed method.

The proposed method consists of a learning-based probabilistic imaging step and random walk-based boundary determination step. All the images, namely, training and test images, are denoised before feature extraction of each pixel. A hybrid classifier is used after feature fusion to obtain the probability of the test image, which indicates the likelihood of the pixel to belong to the liver. Automatic random walk-based refinement is applied to achieve the final segmentation result. The flowchart of the proposed method is shown in Fig 1.

### 2.1 Learning based probabilistic image

Given a target image *T* ∈ *R*^*H*×*W*^, the liver segmentation problem is formulated by assigning each pixel *x* ∈ **T** a label *l* ∈ {0, 1} with liver label *l* = 1 and background label *l* = 0. Here, the probability of each pixel belonging to a liver *p*(*l* = 1|*x*) is estimated with a feature-learning-based method.

#### 2.1.1 Pixel representation

Pixel representation is achieved by applying window-based feature extraction. The values of a larger spatial neighborhood (feature window **P**) with size of *h*×*w* are used to describe the center pixel *x*. The texture features achieve satisfying results for medical images in various tasks. In this study, four representative texture features, namely, local binary pattern (LBP) [20,21], gray level co-occurrence matrix (GLCM) [22], Haar [23], and histogram of oriented gradient (HOG) [24], were selected.

(A) LBP

LBP is the non-parametric operator that describes the local spatial structure of images and is invariant to illumination change with fast calculation. The value of the center pixel *x* is used as a threshold and compared with spatial neighborhoods to obtain a binary code for texture feature description. After defining a neighborhood radius *r*, *N* pixels of radius *r* around *x* are processed to construct the texture feature. Given the intensity *P*_*x*_ of the center pixel *x* and *P*_*n*_(*n* = 1, 2, …, *N*) of spatial neighborhoods, we obtain a binary pattern by comparing *P*_*n*_ with *P*_*x*_ clockwise or counter-clockwise. Each digit of the binary pattern is expressed as

$$s(n)=\{\begin{array}{cc} 1 & P_{n}\geq P_{x}\\ 0 & P_{n}<P_{x} \end{array}$$ (1)

LBP is achieved by converting the binary pattern to a decimal number:

$$LBP(x)=\sum_{n=0}^{N}s(n)\cdot 2^{n}$$ (2)

The LBP texture feature for *x* is shown as follows

$$f_{LBP}\left(x\right)={\left[LBP\left(x_{1}\right),LBP\left(x_{2}\right),\ldots ,LBP\left(x_{h\times w}\right)\right]}^{T}$$ (3)

where *x*_*j*_,*j* ∈[1,*h*×*w*] is the spatial neighborhood of *x* in the feature window.

(B) GLCM

The GLCM consists of the distance and angle between different pixels and is used to extract second-order statistical texture features. Comprehensive data, namely, direction, distance, variation range, and speed, are expressed by the relativity between two intensities with a certain distance and direction. In the feature window, GLCM calculates the probability *p*(*a*,*b*|*d*,*θ*) that the intensity value *a* occurs with other intensity value *b* in a specific spatial distance *d* and direction *θ*. Levels *N* (*a*,*b* ∈ *N*) in an image determines the size of the GLCM (*N* × *N*). A number of GLCMs are produced according to different values of *d* and *θ*. Twelve textural features are used for each GLCM measure the characteristics of texture statistics. These features are energy, contrast, correlation, homogeneity, entropy, autocorrelation, dissimilarity, cluster shade, cluster tendency, maximum probability [25], statistics variance, and sum mean [26]. For each *x* in the center of feature window, the GLCM feature is constructed by the intensity value in the feature window and shown as Eq 4

$$f_{GLCM}\left(x\right)={\left[f_{1}^{d_{j},\theta_{j}},f_{2}^{d_{j},\theta_{j}},\ldots ,f_{12}^{d_{j},\theta_{j}}\right]}^{T}$$ (4)

where $f_{1}^{d_{j},\theta_{j}},f_{2}^{d_{j},\theta_{j}},\ldots ,\text{and }f_{12}^{d_{j},\theta_{j}}$ are the 12 textural feature statistics from one GLCM with distance *d*_*j*_ and direction *θ*_*j*_.

(C) Haar

Haar as an appearance feature is used because of its computational efficiency using integral images [27]. Haar consists of four sets of features, namely, edge, line, center-surround and special diagonal line features. All features are obtained by 15 feature filters with white and black rectangles of specific arrangement [28]. Each feature is scalar and obtained by subtracting the sum of pixels under the white rectangle from the sum of pixels under the black rectangle. For each *x* in the center of the feature window, Haar features are constructed based on the feature filter and intensity value in the feature window and are shows as Eq 5 as follows:

$$f_{Haar}\left(x\right)={\left[f_{1}^{},f_{2}^{},\ldots ,f_{15}^{}\right]}^{T}$$ (5)

where *f*_1_,*f*_2_,…, and *f*_15_ are Haar features with 15 feature filters.

(D) HOG

HOG [29] is an effective appearance feature and complementary to Haar features to collaboratively improve performance. Direction density distribution of the gradient or edge in HOG impressively describes the local appearance and shape. Gradient values *G* and directions *θ* are calculated in the horizontal *G*_*x*_ and vertical *G*_*y*_ directions of the entire image:

$$G=\sqrt{G_{x}^{2}+G_{y}^{2}},\;\theta =\text{arctan}(\frac{G_{y}}{G_{x}})$$ (6)

HOG feature is further represented as follows:

$$f_{HOG}\left(x\right)=\frac{\sum_{j\in P}G(j)T(j)}{\sum_{j\in P}G(j)},\;T(j)=\{\begin{array}{cc} 1 & \theta (j)\in \theta_{k}\\ 0 & else \end{array}$$ (7)

where *j* is the location of the pixel in the feature window, and *θ*_*k*_ is the *k*^th^ angular bins.

#### 2.1.2 Feature fusion

The texture feature is extracted for a pixel by concatenating four features (LBP, GLCM, Haar and HOG) and representing as follows:

$$f\left(x\right)=\left[f_{LBP}\left(x\right)\text{;}f_{GLCM}\left(x\right)\text{;}f_{Haar}\left(x\right)\text{;}f_{HOG}\left(x\right)\right]$$ (8)

The high dimensionality of the concatenated features may result in information redundancy and high computational cost. Principle component analysis (PCA) is used by measuring the correlations between elements to select more useful information and improve performance. Assuming that **F** = [**f**_1_,**f**_2_,⋯,**f**_*N*_] denotes *N* training sample sets and **f**_*n*_,*n* = 1,2,*…*,*N* is the texture feature of *x*_*i*_,*i* = 1,2,*…*,*N*. After the sample sets the centralization $\bar{F}$, the covariance matrix ${\bar{FF}}^{T}$ is created to fine the eigenvector matrix **U** = [**u**_1_,**u**_2_,⋯,**u**_*L*_] and eigenvalue matrix **Σ** = *diag*[*λ*_1_,*λ*_2_,⋯,*λ*_*L*_](*λ*_1_ ≥ *λ*_2_ ≥ ⋯ ≥ *λ*_*L*_). Eigenvectors **u**_*d*_ associated with the first *D* largest eigenvalues are used to form the projection subspace as follows:

$$U_{D}=\left[u_{1},u_{2},\cdots ,u_{D}\right],\;\text{where}\;D<L$$ (9)

The fused texture feature is achieved by projecting the original feature into the PCA subspace:

$$y_{n}=U_{D}^{T}f_{n}$$ (10)

#### 2.1.3 Hybrid classifier for the pixel forecast

A hybrid classifier is used to forecast the probability of a pixel belonging to the liver [30]. Adaboost [31] creates a collection of SVMs [32] as weak classifiers ($h_{t}^{SVM},t=1,2,\ldots ,T$) with adaptive weights *α*_*t*_. Therefore, optimized performance is achieved by avoiding the selection of the optimal parameter in the single classifier. For *N* training sample sets {(**y**_1_,*l*_1_),(**y**_2_,*l*_2_),…,(**y**_*N*_,*l*_*N*_)} with *l*_*n*_ as the label of each training pixel, the weights of training samples are set to $w_{n}^{t}=\frac{1}{N},t=1,2,\ldots ,T$, where *T* is the number of cycles. SVMs with RBF kernel are used as weak classifiers, and the Gaussian width *σ* of RBF decreases in each cycle. The component classifier is trained on the weighted training samples to calculate the training error. For the *t*^th^ cycle, the training error is $e_{t}=\sum_{n=1}^{N}w_{n}^{t}$ when $l_{n}\neq h_{t}^{SVM}\left(y_{n}\right)$. Then the weight is $\alpha_{t}=-\frac{1}{2}\text{ln}\left(\frac{e_{t}}{1-e_{t}}\right)$. A new cycle is started after $w_{n}^{t+1}=\frac{w_{n}^{t}\text{exp}\left\{-\alpha_{t}l_{n}h_{t}^{SVM}\left(y_{n}\right)\right\}}{Z_{t}}$ is updated with *Z*_*t*_ a normalization constant and $\sum_{n=1}^{N}w_{n}^{t+1}=1$. A series of component classifier weights *α*_*t*_ are obtained to construct an SVM-based Adaboost classifier:

$$C\left(y\right)=sign\left(\sum_{t=1}^{T}\alpha_{t}h_{t}^{SVM}\left(y\right)\right)$$ (11)

The probability estimated for the positive class for a two-class problem is calculated as [33]:

$$p\left(l=1|x\right)=\frac{e^{C\left(y\right)}}{1+e^{C\left(y\right)}}$$ (12)

The probabilistic image is formed by the liver-likelihood estimation of each pixel.

### 2.2 Random walks based liver boundary determination

**Fig 2:**
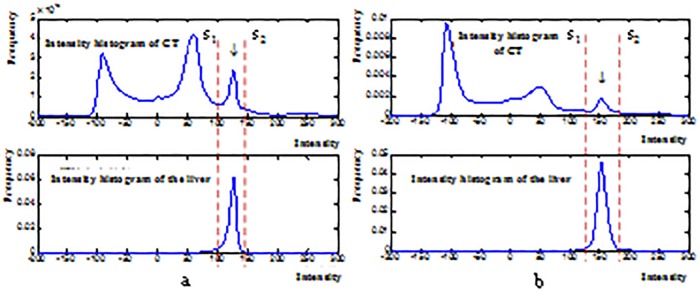
Gray value distributions of two images and the livers. (a) and (b) Distribution of two different datasets.

The probabilistic image provides a prior knowledge without using the relationship of pixels to each other. This process is inappropriate for the direct segmentation of the liver with smooth and continuous boundary. The random walk algorithm [34], which considers the spatial nature of an image, incorporates above prior knowledge to achieve an accurate segmentation result. An adaptive threshold method is adapted in the original image to automatically initialize the seed points required by the random walk. The histograms of each slice of training images are investigated, in which the last peaks in the histograms denote the intensity range of the livers. Fig 2 shows the gray value distribution of one image (upper line) and the liver part (lower line) [35]. Prior knowledge states that the gray level range of the liver is between 125 and 155 [36]. After extracting the peak in this range, the liver is separated from non-liver tissues by determining the thresholds *s*_1_ and *s*_2_. For a contrast-enhanced CT image, *s*_1_ is 3×13HU less than the peak and *s*_2_ is 2×13HU more than the peak [37]. For a test image, the binary images representing the liver and background are expressed as follows:

$$\begin{array}{l} g_{1}(x_{i})=1,\text{ if }(s_{1}<T\left(x_{i}\right)<s_{2})\\ g_{2}(x_{i})=1,\text{ if }(T\left(x_{i}\right)<s_{1}) \end{array}$$ (13)

Erosions are operated to process the binary image *g*_1_ containing liver region and *g*_2_ containing background. The maximum connected regions are found in *g*_1_. The seed points labeled as liver *seed*_*in*_ = 1 and background *seed*_*out*_ = 0 are selected in *g*_1_ and *g*_2_, respectively.

*seed*_*in*_ and *seed*_*out*_ are generated as the seed points of random-walk algorithm, which further determines the liver boundary in the image. The original random-walk algorithm achieves liver segmentation depending only on intensity information and ignores texture feature information. In this paper, the probabilistic image obtained by the texture feature information is combined with the original image to determine the liver boundary. The weight between two neighborhoods is shown in Eq 14:

$$\omega_{ij}=\text{exp}(-\beta ((1-\alpha )||T(i)-T(j)|{|}^{2}+\alpha {\left(p(i)-p(j)\right)}^{2}))$$ (14)

where *i* and *j* represent the indices of pixels in both original image T and probabilistic image **p**; *α* and *β* are adjustment parameters. The liver-likelihood estimation for pixel *x*_*i*_ is achieved by minimizing the following objective function on the basis of the labeled seed points:

$$\underset{x}{\text{arg min}}E=\underset{x}{\text{arg min}}x^{T}Lx=\underset{x}{\text{arg min}}\sum_{e_{ij}\in E}\omega_{ij}{\left(x_{i}^{s}-x_{j}^{s}\right)}^{2}$$ (15)

where

$$L_{ij}=\{\begin{array}{ll} \sum_{j}\omega_{ij} & i=j\\ -\omega_{ij} & i\neq j\text{ and }\left(i,j\right)\text{ is the neighborhood indexes}\\ \text{0} & \text{others} \end{array}$$ (16)

If *x*_*i*_ is greater than 1/2, the label of the pixel is 1, i.e., in the liver region. Otherwise, the label of the pixel is 0, which indicated outside the liver.

## 3. Experimental Results

To validate the proposed method, we test it on two databases: (1) MICCAI 2007 grand challenge data, and (2) clinical cirrhosis data. Both databases were enhanced with contrast agent and scanned in central venous phase. Transversal directions were acquired for CT scans with segmented livers. For the data from MICCAI 2007 grand challenge, the number of slices in each scan varied between 64 and 394 with 512×512 resolution. Pixel spacing varied between 0.55mm and 0.8 mm, whereas inter-slice distance varied from 1 mm to 3 mm. The clinical cirrhosis data used in this study are provided by Chinese Academy of Medical Sciences and Peking Union Medical College, and the study on these data was approved by the institutional ethical review board. The patients involved in our study provide written consent. The number of slices in each scan varied between 71 and 195 with 512×512 resolution.

Fifteen scans are randomly selected from the MICCAI 2007 grand challenge data. And instead of the whole CT scan, only one slice contains the largest liver in each scan is used as the training data. Thus, fifteen slices obtained from MICCAI 2007 grand challenge are implemented as the training data to segment the livers from four data randomly select in MICCAI 2007 grand challenge database and three data in the clinical cirrhosis database.

### 3.1 Objective evaluation

Different coefficients reflecting how well two segmented livers match are computed to compare the performance of the proposed method. Denoting the gold standard segmented manually as A, and the automatically segmented liver as B, we have [38],

Accuracy rate:
  $$ACC=\frac{2\times vol\left(A\cap B\right)}{vol\left(A+B\right)}$$ (17)
Volume overlap:
  $$VOE=1-\frac{vol\left(A\cap B\right)}{vol\left(A\cup B\right)}$$ (18)
Relative volume difference:
  $$RVD=\frac{vol\left(A\backslash B\right)}{vol\left(B\right)}$$ (19)
False negative:
  $$FN=\frac{vol\left(A\backslash B\right)}{vol\left(A\cup B\right)}$$ (20)
False positive:
  $$FP=\frac{vol\left(B\backslash A\right)}{vol\left(A\cup B\right)}$$ (21)
  where *vol*(*) denotes the volume of the region *.

Moreover, three evaluation methods, namely, average surface distance (ASD), root mean squared error (RMSE), and maximum surface distance (MSD), were used to compare different segmentation methods according to the pixel surface distance.

$$ASD(A,B)=\left(\sum_{s_{A}\in S(A)}d(s_{A},S(B))+\sum_{s_{B}\in S(B)}d(s_{B},S(A))\right)/\left(|S(A)|+|S(B)|\right)$$ (22)

$$RMSE(A,B)=\sqrt{\left(\sum_{s_{A}\in S(A)}d^{2}(s_{A},S(B))+\sum_{s_{B}\in S(B)}d^{2}(s_{B},S(A))\right)}/\sqrt{\left(|S(A)|+|S(B)|\right)}$$ (23)

$$MSD(A,B)=\text{max}\left\{\underset{s_{A}\in S(A)}{\text{max}}d^{2}(s_{A},S(B)),\underset{s_{B}\in S(B)}{\text{max}}d^{2}(s_{B},S(A))\right\}$$ (24)

where *S*(*) is the surface voxel of the region *, s_*_ is one of the voxels on the surface of the region *, and $d(s_{{*}_{1}},S({*}_{2}))=\underset{s_{{*}_{2}}\in S({*}_{2})}{\text{min}}||s_{{*}_{1}}-s_{{*}_{2}}||$ is the minimum Euclidean distance between corresponding voxels of two data surfaces. Higher ACCs and lower values of other measures indicate better segmentation.

### 3.2 Training sample selection

**Fig 3:**
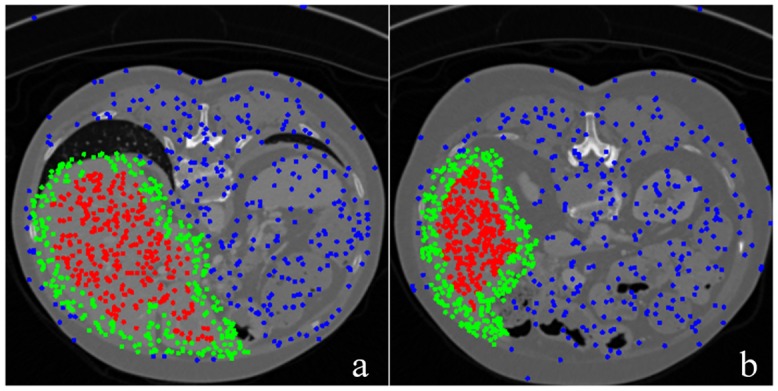
Training sample selection.

Information redundancy is produced when all pixels in the training images are used as training sample. This phenomenon is due to the similar texture features in one image. Moreover, the proportion of positive and negative samples may influence the final classification results. There are total fifteen slices for training as mentioned above. For each training slice, 21000 training samples are randomly selected in three areas, namely, inside (7000 samples), outside (7000 samples), and near the edges of livers (7000 samples). Fig 3 shows the selected training samples.

### 3.3 Parameter selection

**Fig 4:**
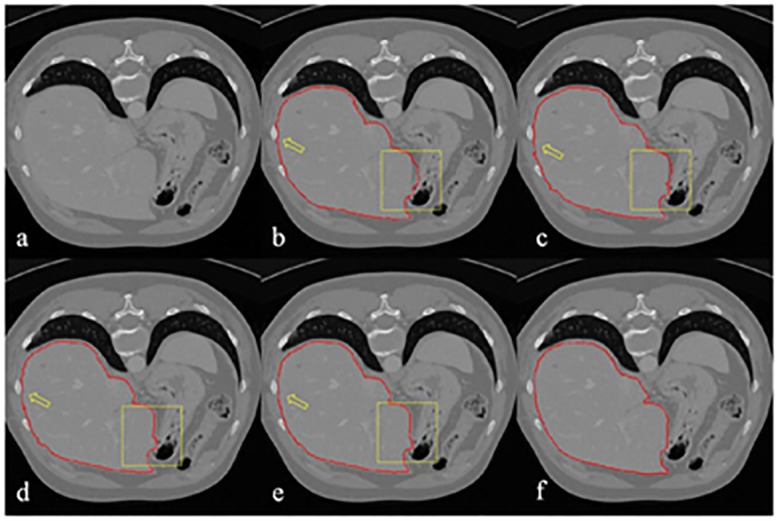
Segmentation results with different *β* parameters.

Parameter *β* of the original random walk segmentation method decides the smoothness of the segmented contour. Fig 4 shows the segmentation results by varying the value of *β*. Fig 4(a) is the original CT slice, and Fig 4(f) is the gold standard. Fig 4(b)–4(e) are the segmentation results obtained when *β* values are is 15, 50, 90, and 150, respectively. The areas pointed out by the yellow arrow and inside the yellow square show that the error of segmentation decreases as *β* increases.

**Fig 5:**
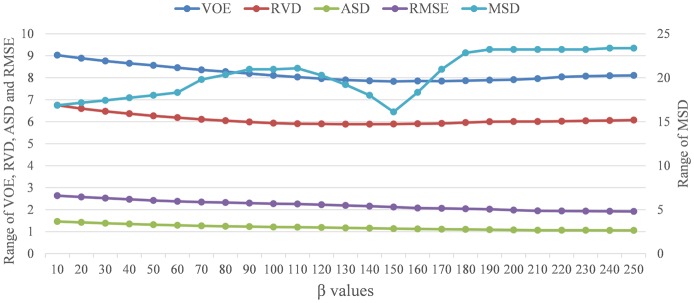
The ranges of VOE, RVD, MSD, ASD and RMSE with different parameter *β*.

Given the optimized parameters, we quantitatively examine the variation trends of objective evaluations with respect to the influences of parameter *β*. Specifically, we perform the liver segmentation with values of parameter *β* from 10 to 250. The changes of the VOE, RVD, ASD, RMSE and MSD with different parameter *β* are shown in Fig 5 and represented with blue, red, green, purple and light blue curves. It is apparent that, for parameter *β* = 150: (1) ASD and RMSE keep decreasing; (2) VOE and MSD achieve minimum; and (3) RVD is near the minimum. Thus, we set parameter *β* = 150 in the subsequent comparative experiment.

### 3.4 Probability image

**Fig 6:**
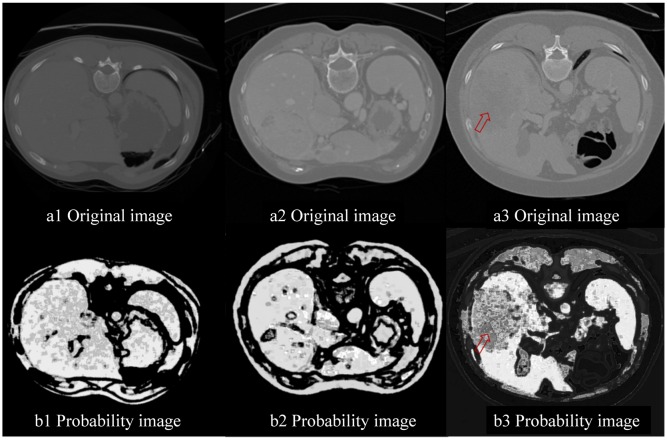
Probability images.

For a test image, the probability image is obtained by classifying each pixel into liver or non-liver based on the extracted texture features. Fig 6 shows the probability images for three different slices of the test image. The liver area is evidently displayed with high brightness and relative clear edge. The tumor area especially pointed out by the red arrow exhibited remarkable difference with the normal liver in the probability image compared with that in the original image. The effectiveness of the extracted features is proved visually. On the other hand, the location of the livers are not exactly same for different patients because of the complexity of anatomical structures. Thus, the texture features are extracted from all the pixels in the images and the probability images are built for all the organs. Since the intensities of stomach, heart and subcostal fat of the rib cage are very similar to the liver, the probability images only is inadequate for liver segmentation. All the organs with different probabilities are shown in Fig 6(b1), 6(b2) and 6(b3). We further increase the robustness of the liver segmentation result using random walk which is improved by combining intensity information and texture feature information.

### 3.5 Comparison on 2D slices of MICCAI data

**Fig 7:**
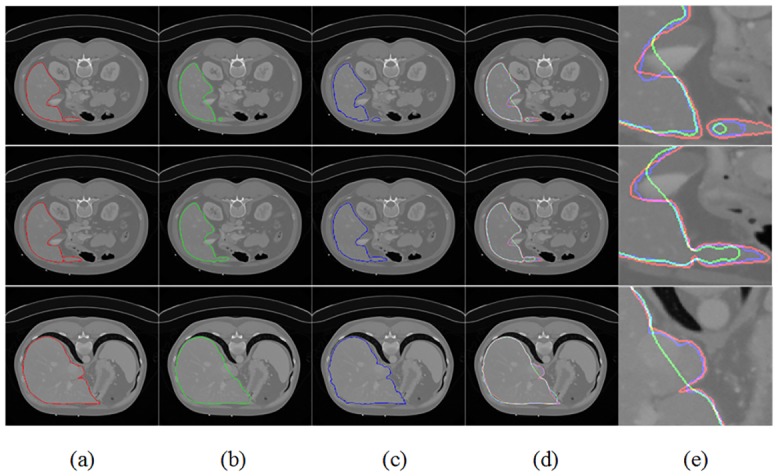
Comparison of results between the gold standard (red curves), RW (green curves) and FLRW (blue curves).

Segmentation results obtained by the proposed method were compared with those obtained by the original random walk method by using the same seed points. Fig 7 displays three different slices in rows with each column indicating: Fig 7(a) the gold standard (red curves), Fig 7(b) the RW segmentation (green curves), and Fig 7(c) the FLRW segmentation (blue curves). Fig 7(e) enlarge the details of Fig 7(d) which present the comparison of three segmentation results. It is clear that the RW evidently resulted in over-segmented or under-segmented results because of the similar intensities between the livers and background. While the FLRW can provide more precise segmentation compared with the original random walk.

Table 1 quantitatively analyzes the ACC, VOE, RVD, FNR and FPR between the RW and FLRW. The average ACC of the FLRW was 95.18%, which was higher than that of the RW. The average VOE, RVD and FPR of FLRW were 9.17%, 9.30% and 0.82%, which were much lower than those of the RW. However, fewer liver voxels were classified as non-liver because of inconspicuous features near the liver edge. The FNR was 8.36% by using FLRW and little more than that of the RW.

**Table 1:** Evaluation of segmentation.

Data	ACC(%)		VOE(%)		RVD(%)		FN(%)		FP(%)	
	RW	FLRW	RW	FLRW	RW	FLRW	RW	FLRW	RW	FLRW
1	92.47	94.17	14.00	11.03	12.34	11.65	10.98	10.43	3.02	0.59
2	93.75	96.03	11.77	7.65	11.77	7.65	8.91	6.74	2.86	0.91
3	94.09	95.35	11.16	8.88	5.43	8.60	5.15	7.92	6.01	0.97
Average	93.44	95.18	12.31	9.17	9.85	9.30	8.35	8.36	3.96	0.82

### 3.6 Segmentation results on 3D data of MICCAI data

**Fig 8:**
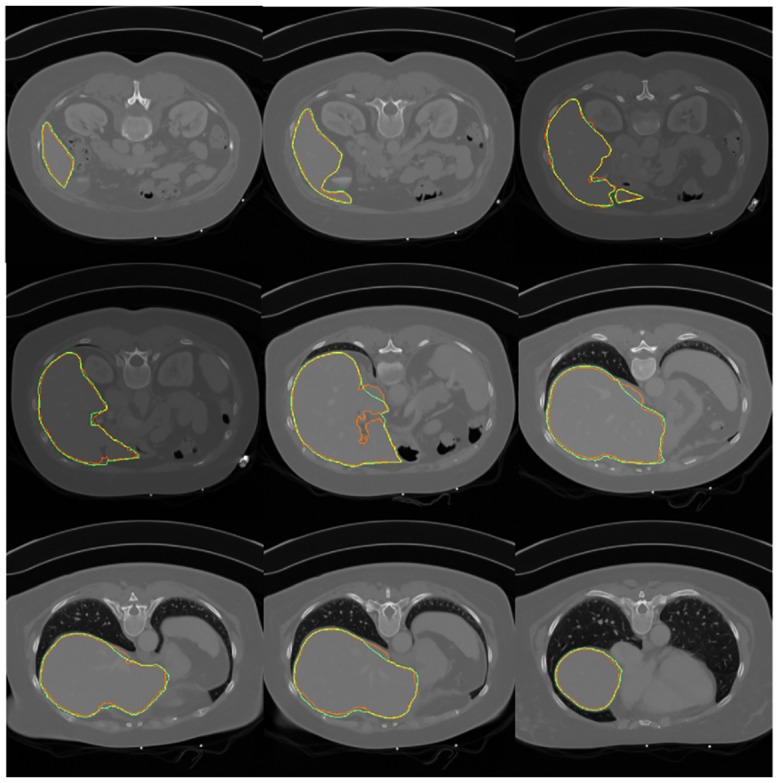
Segmentation results on each slice.

**Fig 9:**
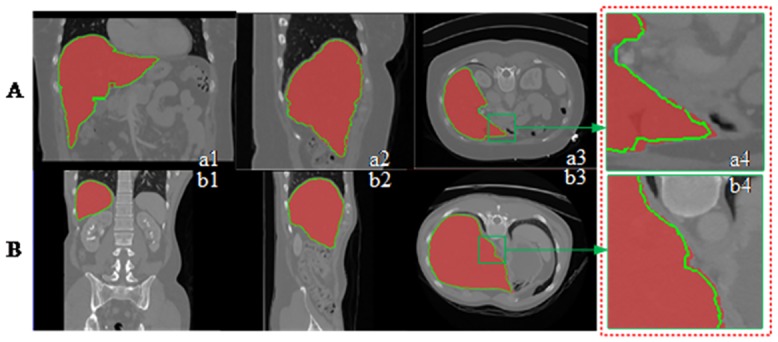
Segmentation results on different directions.

Fig 8 shows the segmentation results on different slices of 3D data. The red and green contours are the segmented edges obtained by the proposed method and random walk. The yellow contours illustrate the overlapping parts. Although the proposed method could not exactly segment the liver edge which has considerable similar intensity with the background, the entire segmentation results still highly conformed with the gold standard. After segmenting all slices in the 3D data, we show the 3D segmentation results in Fig 9 with coronal section, vertical plane, and transverse plane. The segmented edge obtained by the proposed method accurately fitted the liver surface even on the sunken area. This result illustrates the effectiveness of the proposed segmentation method.

**Fig 10:**
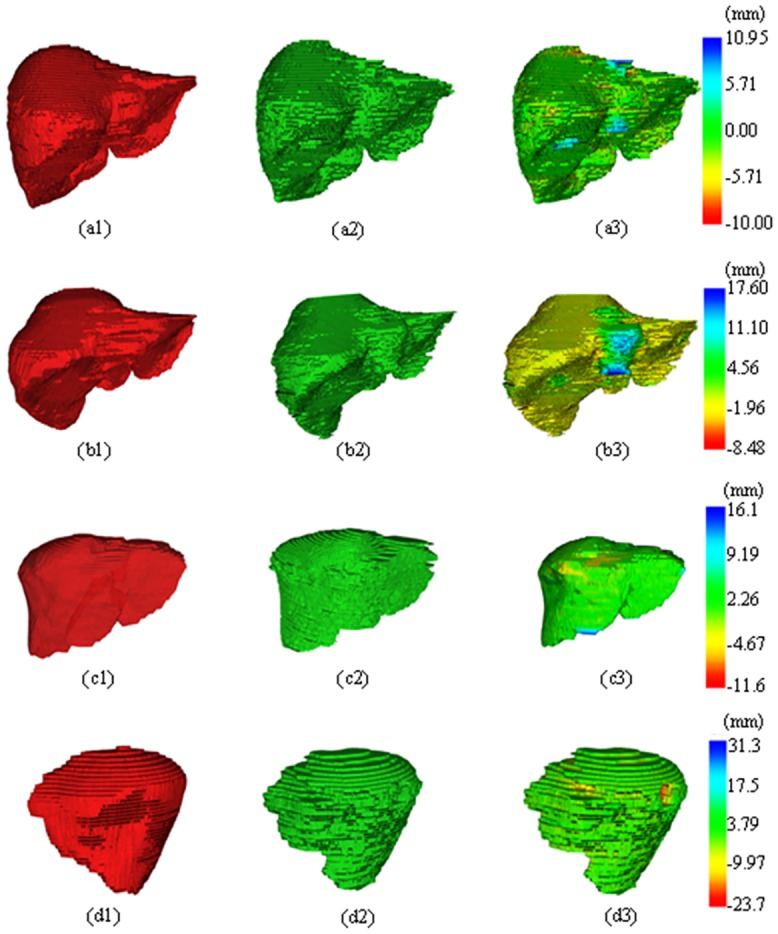
Results of 3D rendering, fusion and distance error map for four test images.

The 3D rendering results are shown in Fig 10, in which (a1~d1) and (a2~d2) are the gold standard and FLRW results, whereas (a3~d3) are the fusion display with 3D surface distance error maps. Most areas are with small distance error even at the corner region. The area connected with the vessel has a large distance error, which should be further improved in future work.

**Fig 11:**
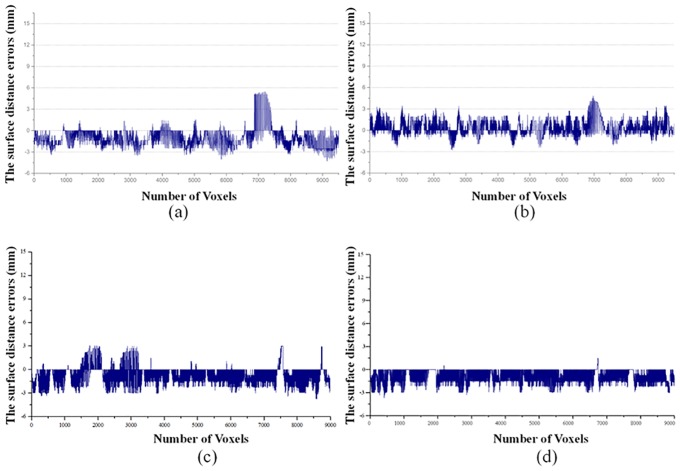
The surface distance error for different voxels in each data: (a) Data 1; (b) Data 2; (c) Data 3; (d) Data 4.

Statistical distribution of the surface distance errors for the different text data are shown in Fig 11. Most of the surface distance errors were in the range of 0mm to 3mm. Surface distance errors of voxels less than 2mm accounted for more than 90% proportion of all voxels. It numerically shows that the FLRW can achieve a relatively accurate segmentation result.

### 3.7 Quantitative error analysis for both databases

Tables 2 and 3 list the quantitative evaluation of segmentation results for four test data in MICCAI 2007 grand challenge database and three data in clinical cirrhosis database. Comprehensive evaluation criteria were provided by the MICCAI 2007 grand challenge and are shown in Table 4. The gold standard as reference is used to calibrate the scores of the test data. The performance of gold standard is 75 out of 100 points (Table 4). The corresponding score for test data is obtained by [7]

$$S=\text{max}\left(100-25\frac{\varepsilon_{i}}{{\bar{\varepsilon }}_{i}},0\right)$$ (25)

where *ε*_*i*_ is the quantitative evaluation value of the test data, and *ε* is the evaluation standard values.

**Table 2:** Quantitative evaluation of MICCAI 2007 grand challenge database.

Data	VOE[%]	RVD[%]	ASD[mm]	RMSE[mm]	MSD[mm]
1	8.80	6.51	1.30	1.98	17.60
2	9.18	5.21	1.57	2.03	10.95
3	7.83	5.89	1.14	2.12	16.12
4	7.63	5.52	0.85	1.87	31.30
Average	8.36	5.78	1.22	2.00	18.99

**Table 3:** Quantitative evaluation of clinical cirrhosis database.

Data	VOE[%]	RVD[%]	ASD[mm]	RMSE[mm]	MSD[mm]
1	11.29	6.4	1.59	3.50	21.00
2	10.82	6.87	1.82	4.17	28.01
3	11.30	6.68	1.94	4.69	29.59
Average	9.43	5.83	1.43	2.55	16.02

**Table 4:** The set five evaluation standard values.

Parameters	VOE[%]	RVD[%]	ASD[mm]	RMSE[mm]	MSD[mm]	score
Standard values	6.4	4.7	1.0	1.8	19	75

Table 5 displays the scores of different methods for comparison. The methods of Heimann [39], Saddi [40], van Rikxoort [41], and Gauriau [42] were performed using the MICCAI 2007 grand challenge database as the proposed FLRW and evaluated with same criteria. Comparative results show that the proposed FLRW was superior to three methods in the total score, and same as or better than the fourth method in ASD and RMSE. Moreover, the FLRW achieved more effective segmentation result with the smallest MSD.

**Table 5:** The score comparisons of different automatic methods with MICCAI database.

Methods	VOE		RVD		ASD		RMSE		MSD		TotalScore
	[%]	Score	[%]	Score	[mm]	Score	[mm]	Score	[mm]	Score	
FLRW	8.36	67	5.78	69	1.22	70	2.00	72	18.99	75	**70.7**
Heimann^[^^39^^]^	7.77	70	1.7	88	1.4	65	3.2	55	30.1	60	**67**
Saddi^[^^40^^]^	8.9	65	1.2	80	1.5	62	3.4	52	29.3	62	**64**
van Rikxoort^[^^41^^]^	12.5	51	1.8	80	2.4	40	4.4	40	32.4	57	**53**
Gauriau^[^^42^^]^	7.2	72	2.6	85	1.3	67	2.6	64	23.1	70	**71.6**

For the clinical cirrhosis database, we also give the scores as shown in Table 6. Although the training slices are selected from the MICCAI 2007 grand challenge database, the results are still competitive by comparing with Table 5.

**Table 6:** The scores of FLRW with clinical cirrhosis database.

Methods	VOE		RVD		ASD		RMSE		MSD		TotalScore
	[%]	Score	[%]	Score	[mm]	Score	[mm]	Score	[mm]	Score	
FLRW	9.43	56	5.83	65	1.43	55	2.55	42	16.02	66	**57.6**

## Conclusion

We propose an automatic segmentation method for the liver based on a feature learning and random walk. Four texture features were extracted and fused to present each pixel in an image. The probability image was further calculated for liver enhancement. Improved random walk with automatically selected seeds was used in the probability image to achieve effective segmentation results. The proposed method was compared with other methods using eight different measures, namely, ASD, RMSE, MSD, ACC, VOE, RVD, FNR, and FPR, in MICCAI 2007 grand challenge database and the clinical cirrhosis database. The calibrated scores of the test data were investigated, and results further proved the effectiveness of the proposed segmentation method.

## References

[1] Haugen AS, Søfteland E, Almeland SK, Sevdalis N, Vonen B, Eide GE, Nortvedt MW, Harthug S. Effect of the world health organization checklist on patient outcomes a stepped wedge cluster randomized controlled trial. Annals of Surgery, 2015, 261(5): 821–828 10.1097/SLA.0000000000000716 24824415

[2] Meng B, Cong W, Xi Y, De Man B, Wang G, Energy Window Optimization for X-ray K-edge Tomographic Imaging, IEEE Transactions on Biomedical Engineering (2015) 10.1109/TBME.2015.2413816 PMC4794431 25794386

[3] Meng B, Cong W, Xi Y, Wang G, Image reconstruction for X-ray K-edge Imaging with Photon Counting Detector, SPIE Developments in X-Ray Tomography IX, 2014

[4] Wei YT, Zhou YB. Research on ct image segmentation of computer-aided liver operation Applied Mechanics and Materials, 2014, 513–517: 3115–3121

[5] Campadelli P, Casiraghi E, Esposito A. Liver segmentation from computed tomography scans: A survey and a new algorithm. Artificial Intelligence in Medicine, 2009, 45(2–3): 185–196 10.1016/j.artmed.2008.07.020 19059767

[6] Mharib AM, Ramli AR, Mashohor S, Mahmood RB. Survey on liver CT image segmentation methods. Artificial Intelligence Review, 2011, 37(2), 83–95

[7] Heimann T, et al Comparison and evaluation of methods for liver segmentation from CT datasets. IEEE Transactions on Medical Imaging, 2009, 28(8), 1251–1265 10.1109/TMI.2009.2013851 19211338

[8] Chi Y, Cashman PMM, Bello F, and Kitney RI. A discussion on the evaluation of a new automatic liver volume segmentation method for specified CT image datasets in Proc. MICCAI Workshop 3-D Segmentation. Clinic: A Grand Challenge, 2007: 167–175

[9] E. van Rikxoort, Arzhaeva Y, B. van Ginneken. Automatic segmentation of the liver in computed tomography scans with voxel classification and atlas matching MICCAI Workshop 3D Segmentation in the Clinic: A Grand Challenge, 2007, pp. 101–108

[10] Dag I, Saka B, Irk D. Galerkin method for the numerical solution of the rlw equation using quintic b-splines. Journal of Computational and Applied Mathematics, 2006, 190: 532–547

[11] Seghers D, Slagmolen P, Lambelin Y, Hermans J, Loeckx D, Maes F, et al Landmark based liver segmentation using local shape and local intensity models MICCAI Workshop 3D Segmentation in the Clinic: A Grand Challenge, 2007, pp. 135–142

[12] Hufnagel H, Pennec X, Ehrhardt J, et al Shape analysis using a point-based statistical shape model built on correspondence probabilities MICCAI Workshop 3D Segmentation in the Clinic: A Grand Challenge, 2007, 4791: 959–967 10.1007/978-3-540-75757-3_116 18051151

[13] Saddi KA, Rousson M, Chefd’hotel C, and Cheriet F, Global-to-local shape matching for liver segmentation in CT imaging, MICCAI Workshop 3D Segmentation in the Clinic: A Grand Challenge, 2007, pp. 207–214

[14] Furukawa D, Shimizu A, and Kobatake H. Automatic liver segmentation based on maximum a posterior probability estimation and level set method MICCAI Workshop 3D Segmentation in the Clinic: A Grand Challenge, 2007, pp. 117–124

[15] Slagmolen P, Elen A, Seghers D, Loeckx D, Maes F, and Haustermans K. Atlas based liver segmentation using nonrigid registration with a B-spline transformation model MICCAI Workshop 3D Segmentation in the Clinic: A Grand Challenge, 2007, pp. 197–206

[16] Linguraru Marius George, Richbourg William J., Watt Jeremy M., Pamulapati Vivek, Summers Ronald M., Liver and Tumor Segmentation and Analysis from CT of Diseased Patients via a Generic Affine Invariant Shape Parameterization and Graph Cuts, Abdominal Imaging Computational and Clinical Applications, 2012, 7029: 198–206

[17] Huang C, Jia F, Li Y, Zhang X, Luo H, Fang C, Fan Y. Automatic Liver Segmentation Based on Shape Constrained Differeomorphic Demons Atlas Registration. In: International Conference on Electronics, Communications and Control 2012, pp. 126–129

[18] Wimmer A, Soza G, Hornegger J. A generic probabilistic active shape model for organ segmentation Medical Image Computing and Computer-Assisted Intervention—MICCAI 2009, 5762: 26–33 10.1007/978-3-642-04271-3_4 20426092

[19] Kainm¨uller D, Lange T, Lamecker H. Shape constrained automatic segmentation of the liver based on a heuristic intensity model MICCAI Workshop 3D Segmentation in the Clinic: A Grand Challenge, 2007, pp. 109–116

[20] Ojala T, Pietik¨ainen M, Harwood D, A comparative study of texture measures with classification based on feature distributions, Pattern Recognition, 1996, 29(1): 51–59

[21] http://www.ee.oulu.fi/mvg/page/lbp_bibliography.

[22] Haralick RM, Shanmugam K, Dinstein Its'hak. Textural Features for Image Classification. IEEE Transactions on Systems, Man, and Cybernetics. 1973, SMC-3 (6): 610–621

[23] Viola P and Jones M. Rapid object detection using a boosted cascade of simple features, Computer Vision and Pattern Recognition, 2001, 1: 511–518.

[24] Dalal N and Triggs B. Histograms of oriented gradients for human detection, IEEE Computer Society Conference on Computer Vision and Pattern Recognition, 2005, 1: 886–893.

[25] Haralick RM, Shanmugam K, Dinstein IH. Textural features for image classification IEEE Transactions on Systems, Man and Cybernetics, 1973, 6: 610–621.

[26] Soh L, Tsatsoulis C. Texture Analysis of SAR Sea Ice Imagery Using Gray Level Co-Occurrence Matrices, IEEE Transactions on Geoscience and Remote Sensing, 1999, 37(2).

[27] Viola PA and Jones MJ. Robust real-time face detection. International Journal of Computer Vision, 2004, 57(2):137–154.

[28] Lienhart R, Maydt J. An extended set of Haar-like features for rapid object detection, International Conference on Image Processing, 2002, 1: I-900—I-903.

[29] Dalal N and Triggs B. Histograms of oriented gradients for human detection IEEE Computer Society Conference on Computer Vision and Pattern Recognition. 2005, 1: 886–893.

[30] Li X, Wang L, Sung E, AdaBoost with SVM-based component classifiers, Engineering Applications of Artificial Intelligence, 2008, 21(5): 785–795

[31] Freund Y, Schapire RE. A decision-theoretic generalization of on-line learning and an application to boosting. Journal of Computer and System Sciences, 1997 55 (1): 119–139.

[32] Vapnik V, 1998 Statistical Learning Theory. Wiley, New York.

[33] Friedman J, Hastie T, and Tibshirani R, Additive logistic regression: a statistical view of boosting, The Annals of Statistics, 2000, 28(2): 337–407

[34] Grady L. Random walks for image segmentation. IEEE Transactions on Pattern Analysis and Machine Intelligence, 2006, 28(11): 1768–1783. 10.1109/TPAMI.2006.233 17063682

[35] Zayane O, Jouini B, Mahjoub MA. Automatic liver segmentation method in CT images. Canadian Journal on Image Processing & Computer Vision, 2011, 2(8): 92–85.

[36] Al-Shaikhli SDS, Yang MY, Rosenhahn B. Automatic 3D Liver Segmentation Using Sparse Representation of Global and Local Image Information via Level Set Formulation, Computer Vision and Pattern Recognition, 2015.

[37] Gao L, Heath DG, Kuszyk BS, Fishman EK, Automatic liver segmentation technique for three-dimensional visualization of CT data, Radiology. 1996, 201(2): 359–64. 10.1148/radiology.201.2.8888223 8888223

[38] Ségonne F, Dale AM, Busa E, Glessner M, Salat D, Hahn HK, Fischl B, A hybrid approach to the skull stripping problem in MRI, Neuroimage, 2004, 22(3):1060–75. 10.1016/j.neuroimage.2004.03.032 15219578

[39] Heimann T, Münzing S, Meinzer HP, Wolf I. A shape-guided deformable model with evolutionary algorithm initialization for 3D soft tissue segmentation Information Processing in Medical Imaging. Springer Berlin Heidelberg, 2007: 1–12. 10.1007/978-3-540-73273-0_1 17633684

[40] Saddi KA, Rousson M, Chefd’hotel C, and Cheriet F, Global-to-local shape matching for liver segmentation in CT imaging, MICCAI Workshop 3D Segmentation in the Clinic: A Grand Challenge, 2007, pp. 207–214

[41] van Rikxoort E, Arzhaeva Y, and van Ginneken B, Automatic segmentation of the liver in computed tomography scans with voxel classification and atlas matching, in Proc MICCAI Workshop 3-D Segmentat. Clinic: A Grand Challenge, 2007: 101–108.

[42] Gauriau R, Cuingnet R, Prevost R, Mory B, Ardon R, Lesage D, Bloch I, A generic, robust and fully-automatic workflow for 3D CT liver segmentation, Proceedings of the 5^th^ Workshop on Computational and Clinical Applications in Abdominal Imaging in conjunction with MICCAI 2013 In Abdominal Imaging. Computation and Clinical Applications: 241–250. Springer Berlin Heidelberg

